# Pregabalin induced visual hallucinations – a rare adverse reaction

**DOI:** 10.1186/s40360-020-0395-6

**Published:** 2020-02-28

**Authors:** Georgios Mousailidis, Basavaraja Papanna, Andrew Salmon, Aung Sein, Qutaiba Al-Hillawi

**Affiliations:** Essex Partnership Trust, Wickford, UK

**Keywords:** Pregabalin, Visual hallucinations, Side effect, Adverse reaction

## Abstract

**Background:**

Pregabalin is an anticonvulsive, analgesic and anxiolytic medication. The typical side effects include dizziness, somnolence and weight gain. Few studies or case reports have demonstrated psychiatric side effects resulting from its use.

**Case presentation:**

We present a patient who suffered visual hallucinations and agitation associated with an increase in pregabalin dose, resolving completely after pregabalin discontinuation.

**Conclusions:**

Acute visual hallucinations should be considered in the clinical spectrum of very rare side effects of pregabalin use, especially at higher doses. Tapered discontinuation of the medication can improve and resolve symptoms.

## Background

Pregabalin is a 3-isobutyl derivative of gamma-amino butyric acid (GABA), which acts as an inhibitory neurotransmitter in the human brain. It is used by clinicians for treating ailments including neuropathic pain, fibromyalgia, generalized anxiety disorder, and partial epileptic seizures. Pregabalin was developed initially as an antiepileptic medication [1]. It binds with high affinity to the alpha-2-delta protein subunit of the P/Q type voltage-gated calcium channels, in presynaptic neurons, and reduces the release of excitatory neurotransmitters in the central nervous system. Pregabalin decreases the concentration of various neurotransmitters, including noradrenaline, glutamate, dopamine, serotonin, and substance P [1, 2]. Despite the name, it does not bind to GABA receptors [3].

Pregabalin has the potential to cause dependence and addiction because of its anti-glutamatergic properties and although often compared to benzodiazepines and alcohol, it is acknowledged that pregabalin is comparatively less addictive than benzodiazepines [4]. However, research has indicated that the sudden discontinuation of pregabalin can potentially cause withdrawal symptoms, such as nausea, insomnia, diarrhea and headache, suggesting some physical dependence [5].

Pregabalin is generally considered to be an effective, well-tolerated and safe medication however, reported adverse reactions, include somnolence, dizziness and headache [6]. Very few case reports or studies have identified psychiatric symptoms, such as psychosis or visual hallucinations as classical adverse reactions from pregabalin, even in patients who receive significant dosages [1].

Here the authors present a patient suffering from chronic anxiety, who developed visual hallucinations and agitation associated with an increase in pregabalin dose.

## Case presentation

A 36 year-old female patient was under the care of our mental health team with a diagnosis of mixed anxiety and depressive disorder, with emotionally unstable personality disorder traits (emotional dysregulation and sensitivity to abandonment). Her past medical history was unremarkable and she was taking no medications for physical maladies.

Two months prior to referral to our services her general practitioner started her on pregabalin 150 mg once daily for anxiety and in this time she had reported no appreciable side effects. She started attending anxiety management groups and there was some improvement regarding her anxiety symptoms. However, the patient was still complaining of generalized anxiety with significant panic attacks on occasion. Her pregabalin dose was increased to 150 mg twice daily and subsequently, after approximately 2 weeks, to 150 mg three times a day. She was also receiving venlafaxine MR 225 mg in the morning, as well as lorazepam 1–2 mg, as required (max 4 mg/24 h). Lorazepam was seldom utilized and only in severe anxiety attacks.

The increase in pregabalin dose appeared effective in managing her anxiety symptoms; improving her everyday functioning and reducing the number of panic attacks. After 3–4 days of receiving pregabalin 150 mg three times a day, she reported visual hallucinations and became increasingly agitated. She described having a ‘foggy state of mind’ and experienced visual hallucinations described as ‘black shadows’ in her visual field mimicking people, animals or objects. She further reported that on occasion the visual hallucinations were well defined and colorful; however, these were infrequent occurrences. There were no signs that the patient was experiencing depersonalization-derealization disorder and she remained alert, fully oriented in time, place and person, had organized speech and her memory was intact. She also maintained insight throughout; clearly distinguishing these hallucinations from reality. She did not report any delusions and there were no hallucinations in other sensory modalities. These visual hallucinations were not associated with any diurnal variation and no identifiable pattern emerged in their occurrence.

Physical examination including full neurological examination was unremarkable. Her visual acuity was within normal limits and ophthalmological examination was unremarkable. Blood test including FBC, Urea & Electrolytes, LFT, TFT, and CRP were normal. Blood exams excluded infection or any metabolic abnormalities. MRI head and EEG conducted 2 days after symptom onset showed no pathological findings. There was no previous history of psychotic symptoms or family history for psychosis. The patient reported lifetime abstinence from any elicit substances, she was a non-smoker and there was no history of alcohol abuse. We had no reasons to believe that the patient might had been tampering with pregabalin dose. Her sleep pattern remained normal and her food intake was the same as before the onset of visual hallucinations.

No other side effects, signs or symptoms were reported apart from visual hallucinations and agitation. The patient herself attributed these symptoms to the increase in pregabalin dose and she requested immediate cessation of pregabalin. Gradual discontinuation of pregabalin was agreed with her and stopped completely after 2 weeks. Visual hallucinations and agitation ceased completely after 5–6 days of pregabalin dose reduction and did not recur. Venlafaxine MR 225 mg in the morning was continued unchanged throughout and as required lorazepam was used to manage agitation symptoms effectively during pregabalin dose reduction. The patient was followed up for almost 1 year after pregabalin cessation and she did not report any visual hallucinations.

## Discussion and conclusions

Pregabalin is considered to be an efficacious medication, which has anticonvulsant, analgesic and anxiolytic properties. Patients receiving it, may experience central nervous system side effects; these are usually mild and of short duration [1]. Pregabalin is increasingly being employed by neurologists, psychiatrists and general practitioners as an effective treatment option for the management of neuropathic pain as seen in its increased prescribing frequency [7].

A systematic review of randomized controlled trials, documented the most common side effects of pregabalin including dizziness, vertigo, incoordination, balance disorder, ataxia, diplopia, blurred vision, tremor, drowsiness, confusion, disturbance of attention, euphoria, disorganized thinking and fatigue [8]. Adverse reactions occur more often at high doses and pregabalin has been associated with psychosis, hallucinations and other neuropsychiatric symptoms when titrated rapidly [9, 10]. In contrast to these studies which suggest that visual hallucinations from pregabalin are mainly attributed to rapid titration, our study indicates that this side effect may be dose depended and can appear in cases of slow titration as well, as happened in our case.

Psychotic symptoms caused by antiepileptic drugs (AEDs) are infrequent; however, AEDs have been associated with the provocation of psychosis or worsening of pre-existing symptoms [11]. Furthermore, paradoxical consequences of some anticonvulsant medications have been associated with concurrent intoxication or overdose; these have also been reported at therapeutic doses, including a patient treated with gabapentin in schizophrenia which resulted in worsening psychosis [12, 13].

Acute onset of delirium, particularly hyperactive delirium has been associated with pregabalin in case reports. Even short duration low doses of pregabalin (75 and 150 mg for 3–7 days) have the potential to initiate hyperactive delirium [14, 15]. A further study showed the occurrence of insidious hypoactive delirium in a patient receiving just 100 mg of pregabalin [16].

Pregabalin has no significant drug interactions and is completely absorbed when taken orally with a linear dose-absorption relationship [17, 18]. Pregabalin has a half-life of 6 h, independent of dose and administration frequency with renal elimination expected to be complete after 30 h (5 half-lives). Renal function is therefore a consideration in dosing pregabalin.

There is a case report that attributes a patient’s first episode of psychosis to rapid pregabalin discontinuation. The patient was receiving a relatively high dose of pregabalin and after cessation they displayed withdrawal symptoms including auditory hallucinations, paranoid ideas and mutism. He also displayed self-harming behaviors and attempted suicide. He was given antipsychotic medication and the symptoms resolved over a 3 week period and did not recur [19].

Pregabalin as a medication can cause dependence, which on some occasions can lead to abuse [20]. Norgaard et al., reported a 38 year-old man who was abusing pregabalin by self-administering 8.4 g of pregabalin daily and after 36 h of pregabalin discontinuation, he reported auditory hallucinations and suicidal ideation with associated sweating and tachycardia. Pregabalin was reintroduced at 600 mg daily alongside chlordiazepoxide and quetiapine. Acute symptoms resolved within 48 h, but the psychotic symptoms persisted until several weeks after pregabalin cessation [21]. The underlying mechanism causing psychotic symptoms after rapid discontinuation of pregabalin is poorly understood and likely to be multifactorial. Benzodiazepines with known GABAergic activity have been associated with cases of psychosis post withdrawal and so this class of receptors have been implicated [22].

There are a number of studies on the use of pregabalin for treating other psychiatric disorders (excluding anxiety), especially substance dependence. A placebo-controlled study reported that pregabalin was superior compared to placebo for the treatment of benzodiazepine withdrawal symptoms, although the difference was not statistically significant [23]. A review regarding use of pregabalin in treating alcohol dependence, reported that findings regarding its effectiveness at 150-450 mg for alcohol withdrawal syndrome are inconsistent, but that it may be effective for relapse avoidance [24]. There are also case reports describing patients with Charles Bonnet syndrome who responded to treatment with pregabalin [25].

Any instance of acute onset psychotic symptoms in an otherwise healthy individual, with no previous history of psychosis, should be extensively investigated to excluding underlying pathology. However, adverse reactions to medications should be a consideration as part of this workup. Our patient suffered from visual hallucinations and agitation after an increase in pregabalin dose. The absence of any abnormality in physical examination, MRI brain, EEG and blood tests coupled with the resolution of these symptoms after pregabalin dose reduction and discontinuation strongly implicate pregabalin as the causative agent in this case. Furthermore, no other changes to medications were made throughout the onset, occurrence or resolution of her psychotic symptoms.

We cannot definitively conclude that this patient’s visual hallucinations were entirely induced by pregabalin, although similar rare occurrences have been reported in the literature. Venlafaxine has been associated with hallucinations and psychotic symptoms previously and so the potential for a synergistic effect with pregabalin cannot be ruled out and further research is needed [26, 27]. We have performed a formal assessment of the likelihood the adverse event was caused by pregabalin, using the Naranjo algorithm and the score was 7, which shows that this is a probable adverse drug reaction due to pregabalin. This adverse drug reaction is listed in the medication label.

The pathophysiological mechanisms causing visual hallucinations in our case are unclear. Despite pregabalin being a GABA analogue, it does not interact with GABA receptors. Pregabalin binds to the alpha2-delta auxiliary subunit of presynapticvoltage-gated calcium channels in the central nervous system. Potent binding at this site attenuates depolarization-induced calcium influx at nerve terminals, witha subsequent reduction in the release of excitatory neurotransmitters, including glutamate, noradrenaline, and substance P. In predisposed individuals, an increase of pregabalin plasma levels might result in abnormal over-synchronization of inhibitory systems and thus significant reduction of glutamate levels [28]. According to glutamatergic theories of schizophrenia, glutamate dysfunction, for example a significant reduction of glutamate levels caused by pregabalin could be the cause of hallucinations and other psychotic symptoms [29]. More data is needed to understand the pathophysiology of pregabalin induced hallucinations and psychotic symptoms.

Pregabalin is considered to be an effective medication with an acceptable side effect profile and its use has increased greatly in recent years by neurologists, psychiatrists and general practitioners. Clinicians should keep in mind that high doses may rarely, cause symptoms of psychosis, acute confusional state or paranoid delusions. Rapid discontinuation has also been associated with onset of psychotic symptoms; the underlying mechanism in both instances is unclear.

Acute visual hallucinations should be considered in the clinical spectrum of very rare side effects of pregabalin use, especially at higher doses. Tapered discontinuation of the medication has improved and resolved symptoms.

Cessation of most medication at the first sign of an adverse reaction will likely result in symptom resolution. Further studies are needed to clarify the precise mechanism that causes visual hallucinations and other psychotic symptoms induced by pregabalin.

## Data Availability

Only accessible in this manuscript.

## Abbreviations

AEDs: Antiepileptic drugs
CRP: C-reactive protein
EEG: Electroencephalogram
FBC: Full blood count
GABA: Gamma-aminobutyric acid
LFT: Liver function tests
MRI: Magnetic resonance imaging
TFT: Thyroid function tests
